# Adverse effects of antiseizure medications: a review of the impact of pharmacogenetics and drugs interactions in clinical practice

**DOI:** 10.3389/fphar.2025.1584566

**Published:** 2025-07-10

**Authors:** Michela De Bellis, Giuseppe d’Orsi, Egidio Maria Rubino, Claudia Arigliano, Massimo Carella, Vittorio Sciruicchio, Antonella Liantonio, Annamaria De Luca, Paola Imbrici

**Affiliations:** ^1^ Department of Pharmacy - Drug Sciences, University of Bari “Aldo Moro”, Bari, Italy; ^2^ Neurology Unit, Epilepsy Center, IRCCS Casa Sollievo della Sofferenza, San Giovanni Rotondo, Foggia, Italy; ^3^ Children Epilepsy and EEG Center, San Paolo Hospital, Bari, Italy

**Keywords:** epilepsy, antiseizure medications, adverse drug reactions, pharmacogenetics, drug interactions

## Abstract

Epilepsy is a chronic and debilitating neurological disorder characterized by the occurrence of spontaneous and recurrent seizures. Despite the availability of several antiseizure medications (ASMs), people with epilepsy often experience drug resistance and adverse effects. This narrative review provides an overview of the main adverse drug reactions (ADR) caused by ASMs, including neurological, metabolic, skin reactions and drug failure, and of the underlying molecular mechanisms. Given the critical contribution of pharmacogenomics and drug-drug interactions to the occurrence of some ADRs, we provide examples of the role of major allelic variations identified in genes encoding for molecules involved in the pharmacokinetics, pharmacodynamics and immune system and emphasize the activity of ASMs as inhibitors or inducers of metabolic enzymes. Improved knowledge of the benefit-risk profile of drugs, also through enhanced pharmacovigilance activity and following guidelines recommendations, could implement patients care avoiding ADRs and favoring a beneficial personalized medicine particularly in vulnerable patients as children, elderly people and pregnant women.

## 1 Challenges in epilepsy treatment

Epilepsy is a complex neurological disorder that can be acquired because of brain injury from trauma, stroke, infections, tumors, or can occur as a consequence of a genetic mutation in proteins controlling brain excitability including ion channels, synaptic proteins or neurotransmitter receptors (Jerome, 2019). It affects ∼50 million people of all ages worldwide and has a lifetime prevalence of ∼1% (Corrales-Hern et al., 2023; Guerrini et al., 2023).

**FIGURE 1:**
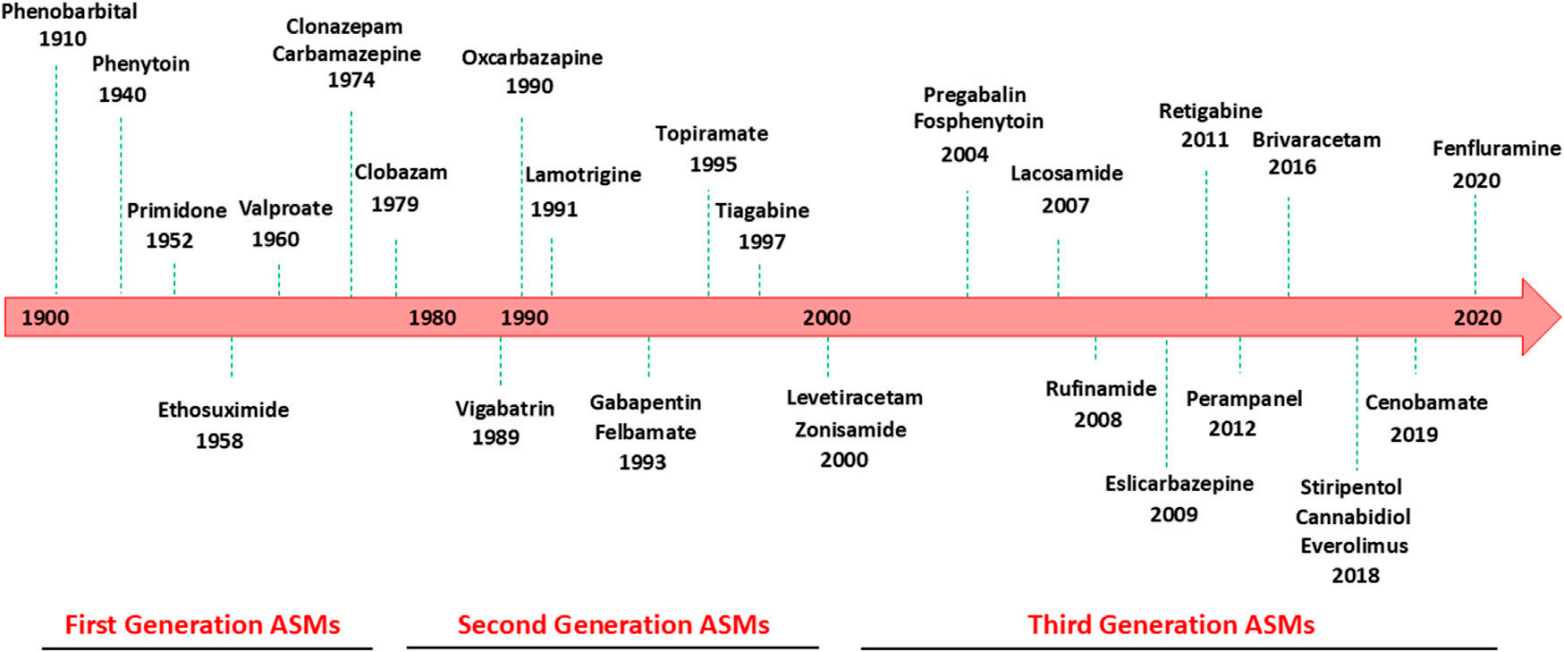
Antiseizure medications available for the treatment of epilepsy and year of market release.

Although the epilepsies are diverse with varying etiologies, they are characterized by repeated, spontaneous epileptic seizures caused by excessive electrical or hypersynchronous neuronal activity in the brain (Wang and Chen, 2019). As such, the primary goal in the treatment and management of epilepsy is the achievement of seizure-free state or, in drug-resistant epilepsies, a significant reduction in seizure frequency, meanwhile minimizing adverse events possibly caused by antiseizure medications (ASMs) and improving patient’s quality of life (Matricardi et al., 2024). Continuous research for new treatments with improved benefit-risk profile resulted in approval of ∼30 ASMs in the US and EU since 1990, classified as first, second and third generation of drugs (Figure 1) (Löscher and Klein, 2021; Pong et al., 2023). Carbamazepine and sodium valproate are the most widely used first generation ASMs whereas new generation of ASMs includes lamotrigine, levetiracetam, and topiramate (Perucca et al., 2020; Gunasekera et al., 2023). Since 2018, new ASMs, cannabidiol, everolimus, cenobamate, and fenfluramine, have been introduced for the treatment of specific populations affected by drug-resistant epilepsy, such as Dravet syndrome (Figure 1) (Guerrini et al., 2024). Some ASMs have additional FDA approved indications besides epilepsy such as neuropathic pain, migraine, anxiety, insomnia, bipolar disorder (Rollo et al., 2023).

**FIGURE 2:**
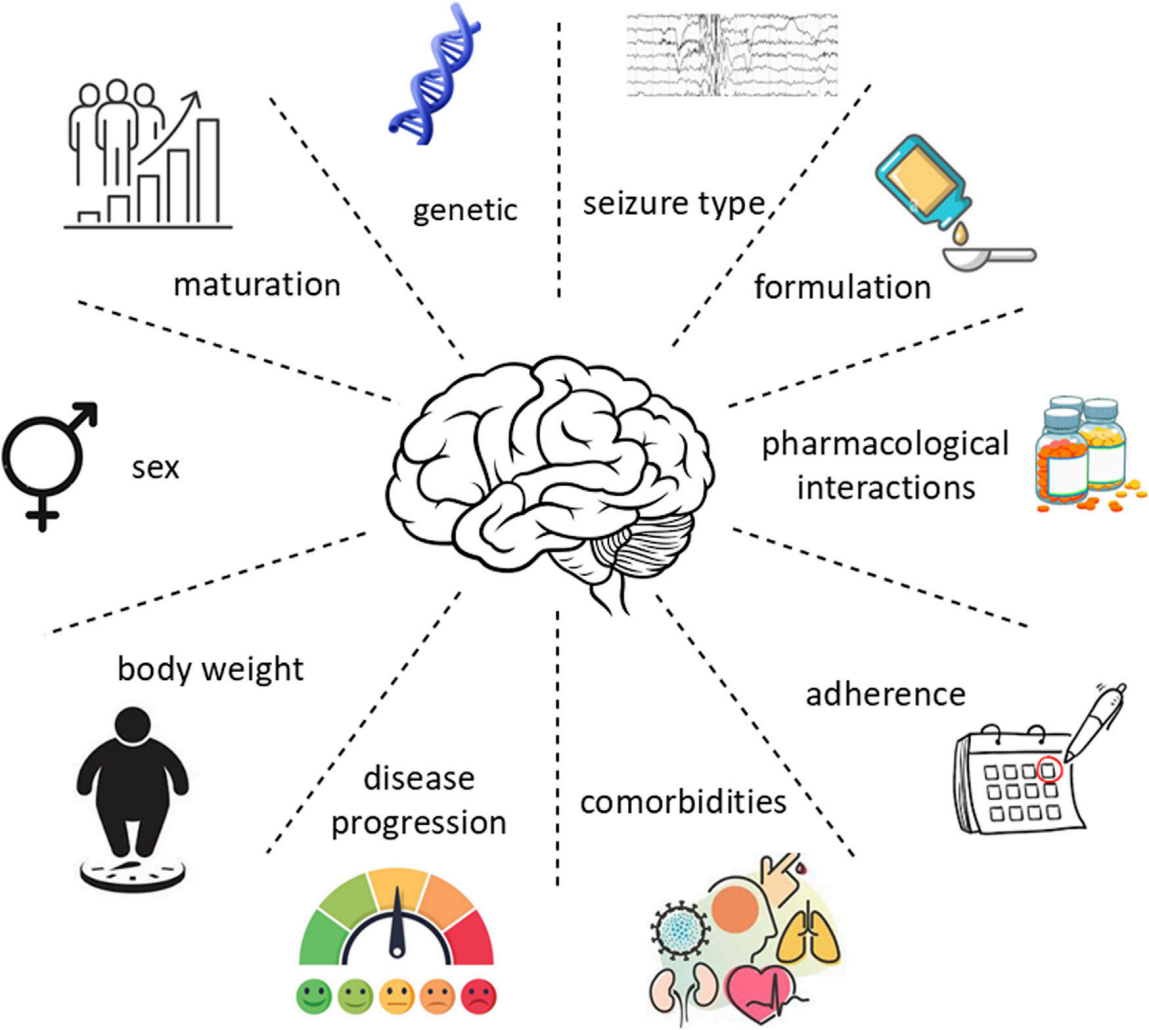
Sources of variability in the response to ASMs.

The appropriate ASM needs to be selected based on the type of seizures, but other individual characteristics such as age, sex, genetics, comorbidities, and concurrent medications should be considered in a personalized risk–benefit analysis (Gunasekera et al., 2023; Perucca, 2021). Once the appropriate ASM has been chosen, the target dosage and the titration rate need to be decided. Depending on the clinical response, adjustments may be needed over the course of the treatment, for example, reduction of the dosage or treatment withdrawal if the patient suffers from adverse effects, or a gradual dosage increase if patients continue to have seizures (Tomson et al., 2023). In some cases, therapeutic drug monitoring is also recommended. Despite different ASMs are available on the market and the arrival of newer drugs, over 30% of patients with epilepsy will continue to have seizures (Fattorusso et al., 2021). In addition, there is large interindividual variability in the response to ASMs in terms of both efficacy and safety (Figure 2). Some early-onset epilepsy syndromes, such as Dravet syndrome, are drug-resistant, and in many cases different ASMs must be administered before having seizures controlled, with increased risk of an adverse drug reaction (ADR) (Corrales-Hern et al., 2023; Guerrini et al., 2024).

ADRs can be dose-dependent or caused by hypersensitivity (Perucca and Gilliam, 2012). Dose-dependent reactions worsen with increasing dose and often occur at the initiation of treatment or are due to drug-drug interactions. Hypersensitivity reactions are often unpredictable and often lead to ASMs treatment discontinuation (Verrotti et al., 2020). Importantly, some of the variation in patients’ response to drugs is related to polymorphisms in genes encoding for proteins involved in drug pharmacokinetics or pharmacodynamics or to individual immune-based susceptibility (Urzì Brancati et al., 2023; Zhao and Meng, 2022). Thus, the identification of subpopulations with either increased or decreased sensitivity to medicines due to genomic factors could provide important information useful to mitigate the risk of side effects and the risk of lack of efficacy in those subpopulations.

## 2 Adverse drug reactions associated with antiseizure medications

Despite numerous attempts to develop safe drugs, ADRs are unavoidable. The different mechanisms of action of ASMs may cause undesired effects; these are mainly neurological and psychiatric but also other organs may be involved. In addition, among the ADRs associated with ASMs, some have a genetic origin or can be attributed to drug-drug interactions, which are generally caused by induction or inhibition of cytochrome P450 (CYP) or other metabolizing enzymes (Urzì Brancati et al., 2023; Zhao and Meng, 2022).

### 2.1 Neurological ADRs

Since ASMs work by modulating neuronal activity in the brain, it is not surprising that most of their adverse effects originate from the central nervous system. The most frequently observed ADRs are: sedation, asthenia, dizziness, coordination disorders (ataxia, dysarthria, diplopia), tremor, cognitive impairment, mood changes, behavioral changes and sexual disorders (loss of libido, impotence) (Perucca and Gilliam, 2012). Their frequency varies according to the type of drug and dose. For example, sedation and cognitive effects are more frequent with barbiturates, benzodiazepines and topiramate. Some of these effects arise from the modulation of γ-amino-butyric-acid (GABA) receptors, glutamate receptors and voltage-gated ion channels by ASMs. Neurological ADRs also depend on the characteristics of the patient; the elderly are more susceptible to cognitive effects and motor coordination disorders, while children are more prone to developing behavioral effects. In addition, the possible presence of medicines used in combination, for example, the co-administration of two or more sodium channel blockers, such as carbamazepine, oxcarbazepine, lamotrigine and lacosamide, involves a greater risk of secondary effects to this mechanism of action, such as a sense of dizziness and coordination disorders (Perucca, 2011).

Among the effects on the central nervous system, the possibility of a paradoxical worsening of epileptic seizures should also be noted. This event can be secondary to an excessive pharmacological load or to the choice of an inappropriate drug for the specific type of epilepsy. For example, the use of carbamazepine and oxcarbazepine in patients with juvenile myoclonic epilepsy or in patients with sodium channels loss-of-function mutations often induces seizure aggravation and may even precipitate a state of malaise (Borowicz-Reutt et al., 2023).

Psychiatric adverse effects may also occur, which include depression, anxiety, irritability, impaired concentration, mood changes, hyperactivity, and, in rare cases, psychosis. Although the newer ASMs are thought to be better tolerated than older drugs, psychiatric adverse effects are common with levetiracetam, topiramate, zonisamide, vigabatrin, and perampanel. Lamotrigine, carbamazepine, valproate, gabapentin, and pregabalin, in contrast, have mood-stabilizing effects in some patients and less frequently cause behavioral or psychiatric effects (Chen et al., 2017). The association between ASMs and a heightened risk of suicidality has been questioned based on case-control and cohort studies and is still controversial (Koseki et al., 2023).

### 2.2 ADRs involving other organs

Other organs and systems may also be affected by ASMs (Fricke-Galindoa et al., 2018). Long-term treatment with ASMs is associated with a four-to five-fold higher probability of osteoporosis (OR = 4.62; CI = 1.40–15.30; *p* = 0.012) and a two-to three-fold increased risk of bone fractures (OR = 2.64; CI = 1.29–5.43; *p* = 0.008) compared to non-ASMs users (Shiek Ahmad et al., 2012). In addition, increased body weight and obesity are common in patients using valproate, carbamazepine, gabapentin, pregabalin, vigabatrin, and perampanel and can lead to serious health consequences associated with obesity and augmented cardiovascular disease risk (Chukwu et al., 2014). In March 2021, the FDA reported that lamotrigine may be associated with a high incidence of cardiac arrhythmias in people with underlying cardiac disease (Christensen et al., 2022). Recently, a large pharmacovigilance study compared the risk of arrhythmia reporting with lamotrigine versus the other ASMs using the World Health Organization (WHO) database Vigibase (ROR = 1.23; 95% CI = 1.14–1.32). A significant association was found for ventricular arrhythmias and cardiac arrest (ROR = 1.63; CI = 1.48–1.80) but not for other arrhythmias. This study, extending FDA alert, underlined the need for cardiac monitoring in people at risk receiving lamotrigine (Orts et al., 2023).

A recent pharmacovigilance study analyzed the most reported ADRs associated with ASMs in the Italian Pharmacovigilance network (Franco et al., 2021). Skin rashes are the most frequent ADR for carbamazepine, lamotrigine, oxcarbazepine and phenobarbital, also classified among the top five ADRs reported for all other medications except for clonazepam (Franco et al., 2021; Farooq et al., 2023) (discussed in paragraph 3.1). Often neglected, gastrointestinal problems, such as nausea, vomiting, abdominal pain, were the most common ADRs associated with ethosuximide possibly because of calcium channels block in the smooth muscle. These effects were also reported at a frequency above 10% with valproic acid, oxcarbazepine, topiramate, and carbamazepine (Franco et al., 2021). This is intriguing considering that patients with epilepsy and autism spectrum disorder often refer to gastrointestinal discomfort as part of the comorbidities associated with epilepsy (Leonard et al., 2022; Riva et al., 2024).

Hyperammonemic encephalopathy (defined as an ammonia level above 80 mcg/dL), sometimes fatal, has been reported following initiation of valproic acid therapy in patients with urea cycle disorders, a group of uncommon genetic abnormalities, particularly ornithine transcarbamylase deficiency (Kazmierski et al., 2021). Valproate-induced acute liver failure and liver-related deaths have been reported in patients with hereditary neurometabolic syndromes caused by mutations in the gene for mitochondrial DNA polymerase gamma (POLG) (e.g., Alpers-Huttenlocher Syndrome) at a higher rate than those without these syndromes (Meseguer et al., 2021).

### 2.3 Chronic ADRs

Some adverse effects of ASMs are subtle and may become apparent only after months or even years of therapy. Examples include hirsutism and gingival hyperplasia induced by phenytoin, shoulder-hand syndrome and Dupuytren’s contraction induced by barbiturates, weight-gain induced by valproate, gabapentin, pregabalin, perampanel and vigabatrin, weight-loss induced by topiramate, zonisamide and felbamate (León Ruiz et al., 2019). Metabolic alterations secondary to enzyme induction (vitamin D deficiency, endocrine disorders, blood lipid abnormalities) may also occur in patients chronically treated with carbamazepine, phenytoin or barbiturates (Perucca and Gilliam, 2012; Fan et al., 2016). Some serious chronic effects led to a drastic reduction in the prescription of certain ASMs, as in the case of irreversible visual field defects induced by vigabatrin that is used to treat tuberous sclerosis complex (Aronica et al., 2023), and abnormal pigmentation of skin, lips, nails and retina induced by retigabine (Brickel et al., 2020; Molimard et al., 2024).

### 2.4 ADRs associated with novel ASMs

New drugs, like fenfluramine, stiripentol and cannabidiol recently approved for the treatment of Dravet Syndrome, Lennox-Gastaut syndrome and other drug-resistant early-onset epilepsies, are well tolerated but not devoid of ADRs (Guerrini et al., 2023; Matricardi et al., 2024). To date, safety information on these drugs is primarily based on data obtained from clinical studies that were analyzed for market authorization (Xu et al., 2024). In the past, valvular heart disease and pulmonary arterial hypertension had been reported with high doses of fenfluramine used for the treatment of obesity in adults. At the lower doses used in children with Dravet syndrome or Lennox-Gastaut syndrome, the most common observed adverse events reported in RCTs for fenfluramine were decreased appetite, diarrhea, fatigue, and weight loss, with no valvular heart disease or pulmonary hypertension observed in any participant (Xu et al., 2024). A recent analysis of the Eudravigilance database suggests that the most common side effects reported for cannabidiol are worsening of epilepsy, increased blood levels of liver enzymes (a sign of hepatic disorder), somnolence, decreased appetite, diarrhoea, fever, and vomiting (Ammendolia et al., 2023). In RCTs, patients treated with stiripentol may experience somnolence (67% vs 23% with placebo), dysarthria (12% vs 0%), and tremors (15% vs 10%), along with gastrointestinal disturbances such as nausea (15% vs 3%) and decreased appetite (46% vs 10%). The adverse effects of these drugs are generally dose-related and can often be managed by appropriate dose adjustments also in consideration of pediatric pharmacokinetics and possible polytherapy (Bacq et al., 2024; Wheless and Weatherspoon, 2025). Cenobamate was approved in 2021 in Europe for the adjunctive treatment of focal-onset seizures in adult patients who have not been adequately controlled with at least two other treatments. In a real-world study, which enrolled 54 patients with a mean age of 27.9 years, the most common adverse events reported for cenobamate are somnolence, dizziness and diplopia (Pietrafusa et al., 2023).

### 2.5 Teratogenesis

Within ADRs, particular attention is given to the teratogenic effects of some ASMs. Most women with epilepsy who become pregnant require continued ASMs therapy for seizure control. The available ASMs have all been shown to increase the likelihood of major congenital malformations (MCMs) (Tomson et al., 2019). The risk of fetal congenital malformation varies in relation to the type of drug, the dose and the number of drugs taken. In addition to careful selection of drug type, the amount of fetal exposure at conception and early pregnancy is probably important for all ASMs, with augmented risk with polytherapy compared to monotherapy (Tomson et al., 2019). Drug concentrations for individual women should be established before conception and maintained throughout pregnancy to prevent worsening of seizures. Interindividual variability supports the use of therapeutic drug monitoring for most ASMs, as significant changes in pharmacokinetics occur for many drugs during pregnancy and post-partum (Pennell, 2016; Imbrici et al., 2011; Ji et al., 2025).

**FIGURE 3:**
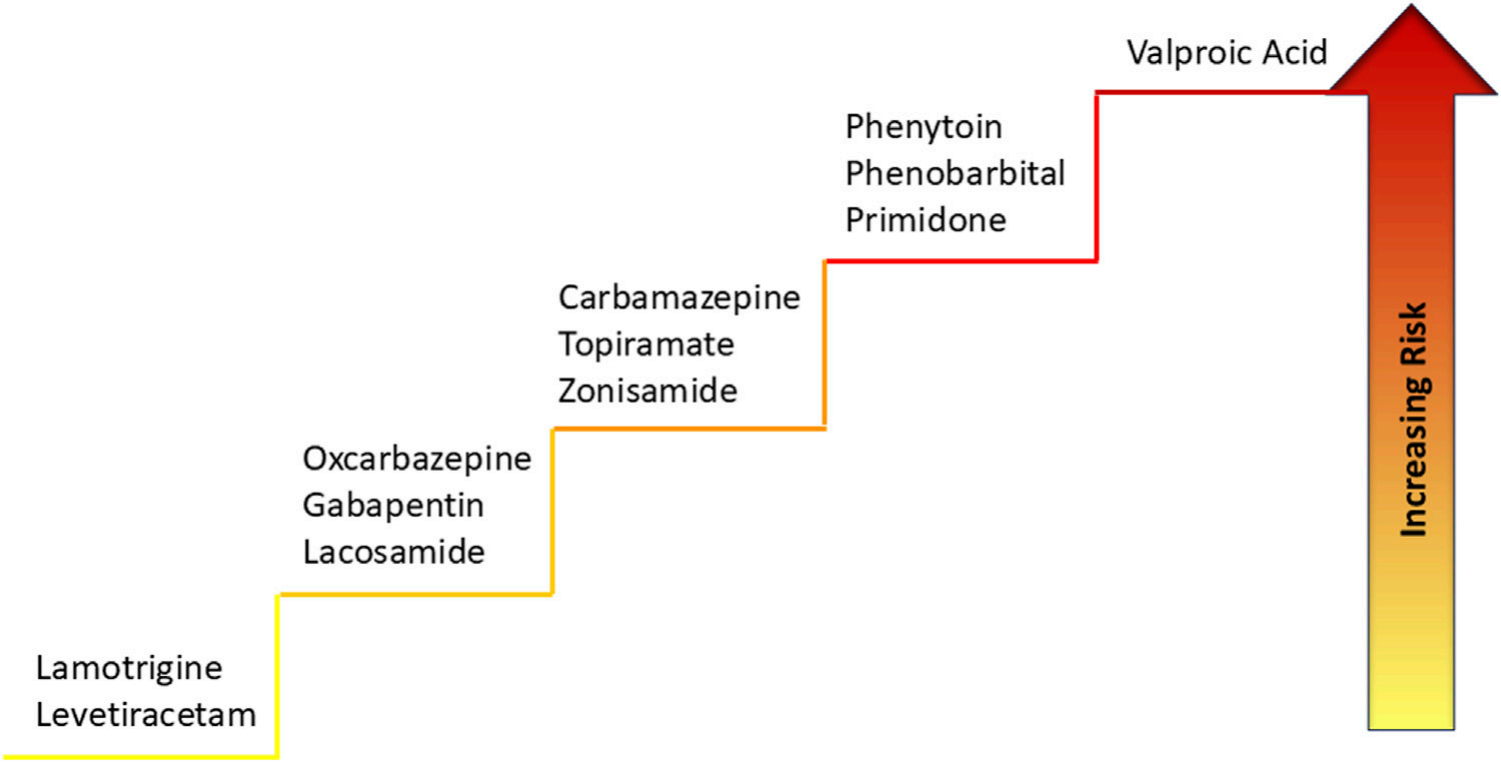
Teratogenic risk profile of ASMs.

In a recent study aimed at comparing the risk of major congenital malformations assessed at 1 year after birth in offspring exposed prenatally to one of eight commonly used ASMs (carbamazepine, lamotrigine, levetiracetam, oxcarbazepine, phenobarbital, phenytoin, topiramate, and valproate), the drug associated with the greatest risk is valproate (Figure 3). Indeed, recent analyses from several large-scale international pregnancy registers and pharmacovigilance databases suggest that *in utero* exposure to valproate during the first trimester is associated with a three-fold higher risk of congenital malformations, commonly neural tube defects (spina bifida) and cardiovascular, orofacial, and digital abnormalities (Tomson et al., 2019; Ji et al., 2025). The use of valproate during the first trimester is also associated with cognitive impairments. In particular, in individuals exposed to valproate, a prevalence of autism spectrum disorder of 6%–15% was observed, a value significantly increased compared to the risk of the background population (Clayton-Smith et al., 2019). In another recent study, the association between ASMs use and fetal disorders was assessed using FAERS data from the first quarter of 2004 to the fourth quarter of 2023. A significant correlation was identified between fetal pathologies and first- and second-generation ASMs, with RORs of 3.8 and 4.9, respectively. Again, valproic acid monotherapy showed the highest correlation with fetal pathologies (ROR = 15.8, PRR = 16.3, IC025 = 3.8) and was uniquely associated with male reproductive toxicity (Ji et al., 2025).

Official guidelines issued by the British Medicines and Healthcare Products Regulatory Agency (MHRA) warn against the use of valproate in female patients unless all other appropriate treatments have failed (Tomson et al., 2018). The risk/benefit ratio of a change in therapy during pregnancy may not be favorable for mother and fetus, and there is evidence of loss of seizure control in women who had suspended valproate use during pregnancy (Tomson et al., 2016). As also recognized by the MHRA and EMA, for some women with epilepsy it may not be possible to stop the valproate and it is necessary to continue treatment during pregnancy, with appropriate specialist evaluation. Carbamazepine may cause neural tube defects and craniofacial anomalies. Fetal hydantoin syndrome is related to the use of phenytoin. Treatment with topiramate during the first trimester of pregnancy is associated with a 10-fold increase in oral clefts risk. Phenobarbital can cause congenital malformations, most often cardiac defects (Battino et al., 2024).

Despite, as previously said, no ASM is known to be entirely safe for the developing fetus (Tomson et al., 2018; Cohen et al., 2024; Battino et al., 2024), lamotrigine, levetiracetam, and oxcarbazepine have the lowest risks of major congenital malformations and may be safer, particularly for cognition, compared with valproate (Stephen et al., 2019). Combining lamotrigine and levetiracetam in patients with idiopathic generalized epilepsy has 60% less risk of teratogenicity than high-dose valproate monotherapy. Whether this association is as effective as valproate for seizure control during pregnancy remains to be determined (Cohen et al., 2024). To date, due to increased awareness, prevalence of MCMs decreased from 6.1% (153 of 2505) during the period 1998 to 2004 to 3.7% (76 of 2054) during the period 2015 to 2022 (Battino et al., 2024).

Overall, although improvements have been made in the clinical management of pregnant women but also of children and elderly suffering from epilepsy, a better knowledge of age-specific pharmacokinetics and pharmacogenomic variability, as well as of interactions among ASMs and between ASMs and other drug classes, would facilitate clinical risk assessment and favor appropriate therapeutic decisions. During pregnancy, changes in the physiological environment of pregnant women may contribute to alter the blood concentration of ASMs thus requiring dosage adjustment to maintain effective control of epilepsy. Similarly, age-related changes in metabolism, renal clearance, gastrointestinal absorption, and serum albumin concentration may require dosage optimization, especially in children and aged people under polytherapy.

## 3 Pharmacogenetics of antiseizure medications (gene-drug interactions)

Advances in genomic technologies have facilitated the discovery of common and rare gene variants and have enhanced our understanding of genetics in epilepsy and other neurologic disorders (Guerrini et al., 2023; Imbrici et al., 2011). Genomic studies also improved our knowledge of the influence of genetics in the mechanisms underlying ASMs toxicity, efficacy and duration of drug action (Cárdenas-Rodríguez et al., 2020). Indeed, genomic factors may play a role in the pathogenesis of both predictable and idiosyncratic ADRs, and may contribute, together with sex, age, ethnicity, type of seizure, suboptimal dosing, to variable response to ASMs to drug resistance (Perucca, 2021). Stratification of individuals based on genotype or phenotype in genomic subpopulations may lead to a significant increase in therapy benefit, decreased risks or both (Bo et al., 2019).

### 3.1 HLA genes and hypersensitivity ADRs

The propensity to develop delayed or non-immediate rare hypersensitivity skin reactions is under genetic control and requires particular attention. Cutaneous hypersensitivity reactions (cHRs) to ASMs occur in 3%–16% of children receiving anticonvulsants (Franco et al., 2021; Błaszczyk et al., 2015; Mori et al., 2021). The most common cutaneous reactions are generalized maculopapular exanthema (MPE) and delayed urticaria that are mild and usually self-limited (Błaszczyk et al., 2015). Other cHRs to ASMs can be severe and life-threatening (severe cutaneous adverse reactions, SCARs) and include drug rash with eosinophilia and systemic symptoms (DRESS), Stevens-Johnson syndrome (SJS), and toxic epidermal necrolysis (TEN) (Franco et al., 2021; da Silva et al., 2016). Potentially fatal dermatological adverse effects secondary to lamotrigine have been highlighted in numerous case reports, especially when lamotrigine is co-administered with valproate. Although it is difficult to predict a severe skin reaction, the drug should be discontinued immediately at the first sign of progressive skin rash (Farooq et al., 2023).

Generally, these reactions appear within a few days after initiating therapy and require prompt treatment discontinuation. Due to the significant cross-reactivity particularly among ASMs with aromatic structure such as carbamazepine, lamotrigine and phenytoin, in patients who have presented these manifestations it is preferable to use alternative drugs with unrelated chemical structure (Mori et al., 2021; Das et al., 2023). Several genetic variations, principally in the genes encoding for human leukocyte antigen HLA-A, HLA-B and cytochrome P450 enzymes, have been significantly associated with a higher risk of developing a SCAR in specific populations (Chang et al., 2020; Alfares et al., 2021). These variations are considered as genomic biomarkers of these ADRs in pharmacogenomics-driven personalized therapy (Rashid et al., 2022).

**FIGURE 4:**
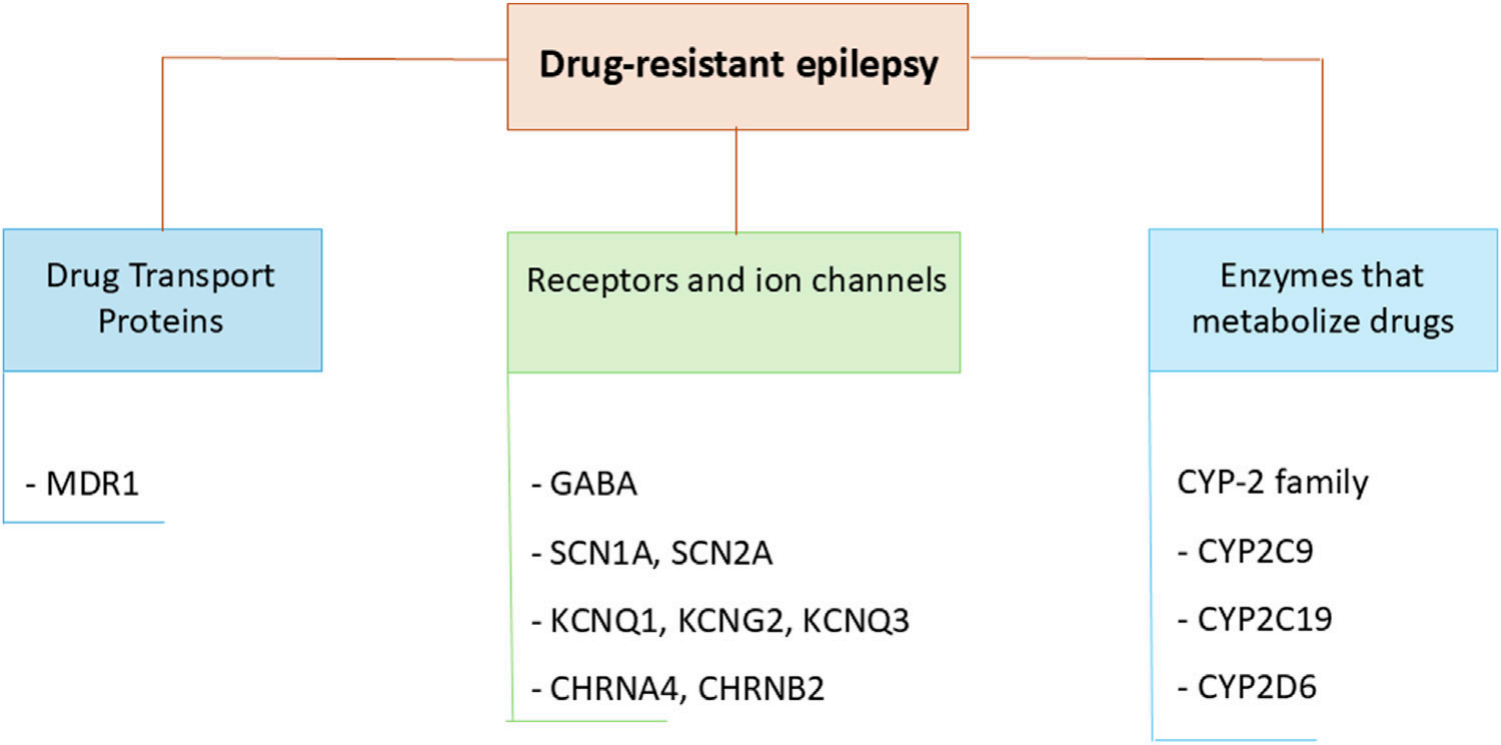
Some polymorphic genes proposed to be involved in drug-resistant epilepsy.

HLA-related SCARs are reported in association with carbamazepine, oxcarbazepine, lamotrigine, phenytoin, phenobarbital, levetiracetam, and valproic acid especially in certain Asian populations, occurring in the first 3 months after initiating therapy (Rashid et al., 2022) (Figure 4). In particular, since 2004, a strong association between the HLA-B*15:02 allele and carbamazepine-induced SJS/TEN has been reported both in Han Chinese and in Thai and Malaysian populations (Tangamornsuksan et al., 2013; Sukasem et al., 2021). In 2007, the US Food and Drug Administration issued a safety warning recommending HLA-B*15:02 screening for people of Asian ancestry before starting carbamazepine and drug avoidance if the test is positive. Subsequent studies from Taiwan, Hong Kong, and Thailand have shown that HLA-B*15:02 screening before starting carbamazepine can significantly reduce the incidence of carbamazepine-induced SJS/TEN (Brandt et al., 2021). Thus, carbamazepine should not be used in an HLA-B*15:02 positive patient. In turn, non-carrier status predicted the absence or low incidence of SCARs induced by the same drug in retrospective post-authorization case-control studies (Brandt et al., 2021).

An increased risk of developing SJS/TEN has been reported in HLA-B*15:02 allele carriers using other ASMs including phenytoin, oxcarbazepine and lamotrigine, although with a 5–10-fold smaller risk compared with carbamazepine (Mullan et al., 2019; Manson et al., 2024).

The polymorphism HLA-A*31:01 has been shown to be a genetic risk factor for SJS/TEN and maculopapular exanthema (MPE) in particular induced by carbamazepine in the Japanese population, as well as in people of European descent (Ozeki et al., 2011; McCormack et al., 2011). Routine testing for HLA-A*31:01, with the aim of reducing the incidence of ADRs in patients prescribed with carbamazepine, has been shown to reduce healthcare costs and is recommended by guidelines (Plumpton et al., 2015). Recently, other alleles, including HLA-B*57:01 and HLA-B*15:11 have also been advised to be associated with carbamazepine-induced SJS/TEN in Europeans (Manson et al., 2024; Mockenhaupt et al., 2019).

Apart from the HLA genes, other genes proposed to affect the risk of SCARs are genes encoding for cytochrome P450 (CYP) drug-metabolizing enzymes. A significant association of CYP2C9*3 with SJS/TEN has been found for phenytoin in patients of southeast Asian ancestry (Chung et al., 2014). This variant has also been associated with decreased clearance of phenytoin (see paragraph 3.2). A recent study demonstrated that the simultaneous testing of CYP2C9*3/HLA-B*13:01/HLA-B*15:02/HLA-B*51:01 increased the sensitivity (from 30.5% to 71.9%) for selecting individuals at risk of developing phenytoin-induced SCAR in Taiwanese populations and confirmed the clinical utility of this strategy in samples from Japan and Thailand (Su et al., 2019).

Increased awareness of SCARs or other ADRs following ASMs use among physicians is of utmost importance for early recognition of symptoms, timely identification and removal of the causative drug, and early intervention to reduce morbidity and mortality. Following emergent genome studies, regulatory agencies as EMA and pharmacogenetics working groups have published evidence-based pharmacogenetics guidelines. These regulations highlight the influence of pharmacogenomics in the assessment of drug safety issues, and explain how to translate the results of evaluations of gene-drug interactions to appropriate pre-treatment recommendations or pharmacotherapy decisions (Guideline on key aspects for the use of pharmacogenomics in the pharmacovigilance of medicinal products EMA/CHMP/281371/2013) (Manson et al., 2024). These guidelines recommend pharmacogenetics testing and therapy modification based on genotype/metabolizer phenotype according to level of evidence (Manson et al., 2024). Examples are the international Clinical Pharmacogenetics Implementation Consortium (CPIC) and the Dutch Pharmacogenetics Working Group (DPWG) guidelines that are annotated by the PharmGKB resource center (Manson et al., 2024). There is not yet consensus on recommendations for gene-drug testing in routine clinical practice for ASMs, and guidelines slightly differ between pharmacogenetics working groups in different countries although the most relevant gene-drug interactions are shared (Manson et al., 2024). Despite increased awareness, significant practical challenges also remain in implementing clinical practices, including the cost and accessibility of genetic testing and the inconsistent adoption of these tests across national healthcare systems.

As example, the Dutch guidelines are reported in Table 1. The DPWG has developed a clinical implementation score and classified recommendations for pre-treatment gene testing in three evidence-based categories (essential, beneficial, or potentially beneficial) to guide physicians in their decision-making prior to initiating a new treatment (Table 1).

**TABLE 1:** Alleles significantly associated with increased risk of severe ADRs from DPWG guidelines.

Genetic biomarker	ADR	ASM	Recommendation	Notes
HLA-B*15:02	SCARs	CBZ, OXC, PHT, LTG	“Beneficial” HLA-B*15:02 genotyping before the start of CBZ, OXC, PHT, LTG in patients of Asian descent, other than Japanese. If possible, choose alternative ASMs in positive patients; if not, advise patients to report any rash immediately	Mostly common in South and East Asian populations except for Japan. In Caucasian or African populations this allele is rare. The risk is 10-fold higher with CBZ for SJS/TEN.
HLA-B*15:11	SCARs	CBZ	“Beneficial” HLA-B*15:11 genotyping before the start of CBZ in East Asian populations. If possible, choose alternative ASMs in positive patients; if not, advise patients to report any rash immediately	Prevalent in East Asian populations
HLA-A*31:01	SCARs	CBZ	“Beneficial” HLA-A*31:01 genotyping before the start of CBZ. If possible, choose alternative ASMs in positive patients; if not, advise patients to report any rash immediately	Prevalent globally, especially in European and Japanese populations

SCARs, severe cutaneous adverse reactions; CBZ, carbamazepine; OXC, oxcarbazepine; PHT, phenytoin; LTG, lamotrigine.

The genotyping of HLA-B*15:02 or CYP2C9 has important limitations and must never substitute for appropriate clinical observation and patient management.

The role of other possible factors in the development of SJS/TEN, and their morbidity, such as ASMs dose, age-related pharmacokinetics, compliance, concomitant medications, comorbidities, and the level of dermatologic monitoring have not been studied in detail and cannot be excluded.

### 3.2 Polymorphisms in genes encoding for proteins involved in pharmacokinetics and pharmacodynamics

Recent studies have identified predictive biomarkers of drug response and of increased ADRs risk among genes encoding enzymes that metabolize drugs, drug transporter proteins, targets of ASMs, namely, among genes involved in the pharmacokinetics and pharmacodynamics of ASMs (Figure 4) (Urzì Brancati et al., 2023; Wolking et al., 2021).

Polymorphisms in genes encoding for CYP and UDP-glucuronosyltransferase (UGT) enzymes may affect ASMs serum concentrations, thus influencing drug response in terms of toxicity and efficacy (Urzì Brancati et al., 2023). Despite different UTG isoenzymes polymorphisms have also been reported, CYP2C9 and CYP2C19 polymorphisms are of major interest and lead to clinical recommendations. Several studies suggest that CYP2C9/2C19 single nucleotide polymorphisms (including CYP2C9*2, CYP2C9*3, CYP2C19*2, and CYP2C19*3) may be relevant in the metabolism and bioavailability of phenytoin, carbamazepine, brivaracetam, clobazam, lacosamide and partially valproate, and may have a clinical impact in patients’ response to therapy (Bo et al., 2019; Ahmed et al., 2021; Alvarado et al., 2023).

For example, phenytoin is primarily metabolized by CYP2C9 and to a lesser extent by CYP2C19 and is characterized by autoinduction (Manson et al., 2024). According to guidelines, based on the metabolic capacity of CYP2C9 genotypes, different metabolizer phenotypes can be distinguished. As polymorphisms in the CYP2C9 gene generally lead to an enzyme with reduced activity, intermediate and poor metabolizers (IMs and PMs) have been identified. In both cases, an increased plasma concentration may lead to an elevated risk of phenytoin side effects such as ataxia, nystagmus, slurred speech, sedation or rash (Table 1). Thus, patients who are known to be IMs or PMs may ultimately require lower doses of phenytoin to maintain similar steady-state concentrations compared to normal metabolizers (Manson et al., 2024). The therapeutic recommendations for phenytoin and dosing adjustment according to CYP2C9 genotype may vary depending on regulatory agencies.

The CYP isoforms mainly responsible for the formation of the major metabolite of lacosamide (O-desmethyl) are CYP3A4, CYP2C9, and CYP2C19. In a recent study, based on CYP2C19 genotypes, patients were stratified in extensive metabolizers (EMs), IMs and PMs. Significantly higher lacosamide concentrations were found in PMs, who also presented the lowest proportion of lacosamide-resistant patients. Thus, when prescribing lacosamide to patients, the CYP2C19 genotype should be considered to optimize drug efficacy and minimize the occurrence of adverse events (Ahn et al., 2022).

In CYP2C19 PMs, the levels of clobazam active metabolite, N-desmethylclobazam, may raise resulting in higher ADR risk. Therefore, in patients known to be CYP2C19 PMs, dosage adjustment is recommended by FDA (Dean, 2019). Furthermore, preliminary studies indicate that polymorphisms in CYP2C19 (such as CYP2C19*2 and CYP2C19*3) may significantly affect stiripentol metabolism. Patients who are PMs can exhibit higher plasma concentrations of stiripentol, which in turn may enhance both its therapeutic efficacy and the risk of adverse events, especially when used in combination with clobazam and valproate. Accordingly, genotyping for CYP2C19 could be beneficial in optimizing stiripentol dosing in clinical practice (Peigné et al., 2018).

Additional genomic biomarkers of variability in drug response are the polymorphisms in drug efflux transporters affecting absorption, distribution and excretion of drugs across various biological membranes, such as the ATP-binding cassette subfamily B member 1 (ABCB1 or MDR1, encoding the P-gp efflux transporter) and ATP-binding cassette subfamily C member 2 (ABCC2 or MRP2) (Bruhn and Cascorbi, 2014). In the brain, P-gp is expressed in astrocytes, endothelial cells, and neurons and overexpression of P-gp in epileptic tissue has been associated with reduced brain concentration and drug resistance to ASMs (Potschka and Brodie, 2012). Several studies reported a correlation between polymorphisms in transporters genes and oxcarbazepine, lamotrigine and lacosamide concentration and patients’ response. However, the significant association between these variants and ASMs is still uncertain (Urzì Brancati et al., 2023).

Changes in pharmacokinetics that can influence the drug plasma concentration occur from childhood to adolescence and adulthood, and further to older age, and in the presence of co-occurring pathologies. Therapeutic drug monitoring may be useful in cases of pharmacokinetic changes (e.g., pregnancy and concomitant use of interacting drugs) and when ASMs toxicity is suspected, thus facilitating optimal dosing in the individual patient (Landmark and Johannessen, 2012).

Polymorphisms in genes coding for ASMs targets such as voltage-gated sodium channels (*SCN1A* and *SCN2A*) or synaptic vesicle protein SV2A, have been also investigated in correlation with the efficacy and toxicity of many ASMs. However, considering that few pharmacogenetic studies in epileptic patients treated with ASMs are present in the literature, the role of these SNPs pharmacodynamics and efficacy of ASMs needs further investigation (Urzì Brancati et al., 2023).

## 4 Drug-drug interactions as a mechanism underlying enhanced toxicity or pharmacoresistance

ASMs are widely used as long-term monotherapy or adjunctive therapy in epilepsy. Co-prescribing of two or more ASMs occurs in about 25% of children and 59.6% of adolescents and adults, typically with more severe types of epilepsy and other indications (Chen et al., 2024). In addition to genetic factors, ASMs are highly susceptible to pharmacokinetic drug-drug interactions that are clinically relevant as these result either in changes in therapeutic effect (augmentation or diminution), or in potentiation of adverse effects (Urzì Brancati et al., 2023; Antanasković and Janković, 2023). This is because many ASMs are substrates, inducers, and/or inhibitors of drug-metabolizing enzymes, transporters or efflux pumps. As mentioned above, the majority of ASMs undergo extensive metabolism, mainly through oxidation by CYP enzymes or glucuronidation by UGTs. Exceptions include levetiracetam and rufinamide which undergo hydrolysis, and gabapentin, pregabalin, and vigabatrin which are excreted unchanged through the kidneys (Antanasković and Janković, 2023).

Carbamazepine, phenobarbital, phenytoin, primidone, among older ASMs, are known to cause strong induction of metabolizing enzymes, such as CYP and UGT, and transporters, whereas valproic acid, cannabidiol and stiripentol cause inhibition, resulting in a decrease or increase, respectively, in the serum concentration of co-prescribed ASMs (Landmark et al., 2023). Accordingly, inducers can reduce the efficacy of co-administered ASMs such as lamotrigine (a UGT substrate), perampanel, and everolimus (CYP3A4/5 substrates). Oxcarbazepine, eslicarbazepine acetate, felbamate, cenobamate, topiramate (at doses ≥200 mg/day), have mixed induction/inhibition properties. Low-interacting drugs include gabapentin, levetiracetam, vigabatrin, pregabalin, lacosamide, lamotrigine, perampanel and fenfluramine (Landmark et al., 2023) (Table 2).

**TABLE 2:** Examples of drug-drug interactions.

Drugs	Action	Main interacting ASMs
Carbamazepine phenytoin phenobarbital primidone	Enzyme inducers, reduce the serum concentration and increase the clearance of co-administered ASMs, with reduction of efficacy of co-administered ASMs	Lamotrigine, valproic acid, ethosuximide, topiramate, zonisamide, clobazam, tiagabine
Valproic acid cannabidiol stiripentol	Enzyme inhibitors, increase the serum concentration and reduce the clearance of co-administered ASMs with enhanced risk of overdose and toxicity	Phenobarbital (sedation), lamotrigine (cutaneous reactions), carbamazepine and carbamazepine 10,11-epoxide (dizziness, ataxia, somnolence), phenytoin (encephalopathy, hypotonia), clobazam (somnolence, hypotonia, irritability)

ADRs, are indicated in brackets.

Enzyme inhibition causes a decrease in the metabolic clearance of the affected drug, the serum concentration of which may increase leading to toxic effects. For example, valproic acid can reduce the clearance of lamotrigine by one-half, leading to an enhanced risk of lamotrigine intoxication and life-threatening hypersensitivity reactions (Yamamoto et al., 2024). Similarly, cannabidiol is an inhibitor of CYP3A4 and CYP2C19 and to a lesser extent of other CYPs and UGT enzymes. Pharmacovigilance data show that the most common drug association in serious ADRs reporting to cannabidiol is between cannabidiol, clobazam and valproic acid, followed by the association of cannabidiol-clobazam-lamotrigine (Ammendolia et al., 2023). Co-administration of cannabidiol with stiripentol induces a slight increase in plasma concentrations of stiripentol, because CYP2C19 is pivotal for stiripentol metabolism. Conversely stiripentol does not affect plasma concentrations of cannabidiol (Morrison et al., 2019). On the other hand, stiripentol acts as a potent inhibitor of CYP1A2, CYP2C19, and CYP3A4. Its use in combination therapy—most notably with clobazam and valproate in Dravet syndrome—has been shown to significantly elevate the plasma concentrations of clobazam and its active metabolite N-desmetilclobazam, thereby increasing both its therapeutic effect and the risk of adverse events such as sedation and drowsiness (Wheless and Weatherspoon, 2025).

A recent study suggested that cenobamate may reduce plasma concentrations of drugs metabolized by CYP3A4/5 and CYP2B6 and may increase plasma concentrations of drugs metabolized by CYP2C19 (Greene et al., 2022). Thus, in clinical practice, when high doses of enzyme inhibitors, such as cannabidiol, valproate or stiripentol, or of inducers, such as carbamazepine, are used to treat childhood epilepsy, it is necessary to adjust the dose of co-administered ASMs.

Also, caution must be taken when an inducer or inhibitor ASM is discontinued, because serum concentrations of the co-administered drug may return to baseline even weeks after the change (Landmark et al., 2023; Yamamoto et al., 2024). Due to unpredictable pharmacokinetic variability and drug interactions with ASMs, therapeutic monitoring of drug serum concentrations can be advisable to warrant adherence and prevent safety issues.

Several drug-drug interactions can occur between ASMs and other drug classes (anticoagulants, oral contraceptives, calcium channel blockers, statins, antidepressants, antipsychotics, antidiabetic drugs, and oral contraceptives, antibiotics, anti-HIV drugs, and immunosupressants) for which a dose adjustment can be required in combination with enzyme-inducing or -inhibiting ASMs (Landmark et al., 2023; Ranzato et al., 2024). For some drugs which are converted to active metabolites, enzyme induction may lead to an increased concentration of the active metabolite and, consequently, an enhancement of clinical effects/risk of toxicity. For example, the induction of cyclophosphamide and thiotepa metabolism by phenytoin can increase, to a clinically significant extent, the exposure to the active metabolites 4-hydroxy-cyclophosphamide and tepa, respectively, requiring a reduction in the dosage of both anticancer drugs (Zaccara and Perucca, 2024). In addition, ASMs metabolisms can be enhanced or inhibited by co-administered drugs of different class; examples are the increase in the serum concentration of carbamazepine by erythromycin and the decrease in the serum concentration of lamotrigine by estrogen-containing contraceptives (Landmark et al., 2023; Sidhu et al., 2006).

Although some drug-drug interactions between ASMs are first investigated *in vitro* or in animal studies upon drug development, the frequency of these ADRs can be clear only in the real world setting. Paying more attention to registration, follow-up, and causality assessment of adverse events when designing both clinical trials and observational studies would help the identification of clinically relevant interactions, which may further be analyzed in terms of pathophysiology.

## 5 Withdrawal syndromes

The occurrence of ADRs, as well as the achievement of sustained seizure freedom, may necessitate the discontinuation of ASMs. However, withdrawal from ASMs can sometimes lead to the development of withdrawal syndromes, as the body has adapted to the presence of the medication and undergoes physiological changes upon its cessation. Withdrawal symptoms can range from mild to severe and may include cognitive impairment (e.g., memory disturbances), increased seizure frequency, mood disturbances such as anxiety and depression, and, in some cases, life-threatening seizures (status epilepticus). Determining the balance of benefits and risks associated with ASMs discontinuation remains a critical challenge for clinicians. To minimize the risk of such complications, a gradual tapering of the drug under close medical supervision is essential. This is particularly important for benzodiazepines and barbiturates, which are associated with a higher risk of withdrawal symptoms and therefore require a slower and carefully managed drug tapering process (Zhang et al., 2004).

## 6 Conclusion

The genetic and phenotypic heterogeneity of epilepsies and the broad spectrum of efficacy, safety, and tolerability related to the ASMs, make the management of affected patients challenging. In the attempt to increase the benefit-risk profile of therapies and to shift toward a personalized medicine in epilepsy (Dinoi et al., 2024; Imbrici et al., 2003), careful knowledge of adverse events of ASMs and their mechanistic basis, as emerged from RCTs as well as pharmacogenetics, post-marketing surveillance and pharmaco-epidemiological studies, is fundamental to tailor the choice of drug and its dosage to the characteristics of the individual patient. In particular, post-marketing surveillance and pharmacogenetics can be essential to detect and explain rare or chronic adverse effects in the real world setting that may not be evident during the pre-marketing clinical phase. Pharmacogenetics testing has become more popular over the last decade, but it is not yet a routine assessment, except in oncology and for few drugs with strong pharmacogenetic associations (Manson et al., 2024). The identification of the gene-drug interaction of CYP2C9 and HLA-B with phenytoin, HLA-A and HLA-B with carbamazepine and HLA-B with oxcarbazepine and lamotrigine has contributed to the development of evidence-based pharmacogenetics guidelines to optimize pharmacotherapy and reduce the risk of severe ADRs. However, genetic testing is far from being cost- and time-effective; pharmacogenetics research findings are sometimes heterogeneous and there is not yet consensus on clinical recommendations (Manson et al., 2024). Future directions point to the combined implementation of pharmacogenetic tests where relevant, therapeutic drug monitoring, and the use of biochemical markers to improve personalized treatment with ASMs. Advances in testing methodologies, such as next-generation sequencing and machine learning approaches, hold promise for identifying novel genetic variants and improving risk-stratification. To fully realize these benefits, it is essential to develop robust infrastructure and provide education and training in the field. Incorporating pharmacogenetic testing into hospital protocols and expanding access to genetic testing across healthcare systems would significantly advance clinical practice.
